# Extensively Drug-Resistant Tuberculosis, Burkina Faso

**DOI:** 10.3201/eid1605.091262

**Published:** 2010-05

**Authors:** Nuccia Saleri, Gisèle Badoum, Martial Ouedraogo, Sary M. Dembélé, Rachel Nacanabo, Victor Bonkoungou, Daniela Cirillo, Gabriele Pinsi, Alberto Matteelli

**Affiliations:** Ministry of Health National Tuberculosis Program, Ouagadougou, Burkina Faso (N. Saleri, S.M. Dembélé, R. Nacanabo, A. Matteelli); University of Brescia, Brescia, Italy (N. Saleri, A. Matteelli); University of Ouagadougou/Yalgado National Hospital, Ouagadougou (G. Badoum, M. Ouedraogo); National Global Fund Coordination, Ouagadougou (V. Bonkoungou); San Raffaele Scientific Institute, Milan, Italy (D. Cirillo); Institute of Microbiology, Brescia (G. Pinsi)

**Keywords:** Tuberculosis, drug resistance, chronic TB, tuberculosis and other mycobacteria, bacteria, Burkina Faso, dispatch

## Abstract

Because data from countries in Africa are limited, we measured the proportion of extensively drug-resistant (XDR) tuberculosis (TB) cases among TB patients in Burkina Faso for whom retreatment was failing. Of 34 patients with multidrug-resistant TB, 2 had an XDR TB strain. Second-line TB drugs should be strictly controlled to prevent further XDR TB increase.

Extensively drug-resistant (XDR) tuberculosis (TB) represents an emerging public health problem worldwide, characterized by alarming fatality rates regardless of patients’ HIV status (*1*,*2*). XDR TB is defined as in vitro resistance to isoniazid and rifampin plus any fluoroquinolone and at least 1 injectable drug (capreomycin, kanamycin, or amikacin).

Since 2006, a total of 49 countries have reported XDR TB (*3*). Data from countries in Africa are scant, with the exception of South Africa, where the prevalence is high, especially among HIV-infected persons (*3*). However, these data may reflect diagnostic limitations rather than the true epidemiologic situation. In Burkina Faso, a low-income country in western Africa, TB prevalence is 226 new cases per 100,000 population (*4*), and multidrug-resistant (MDR) TB is estimated at 2.1% among new patients with smear-positive TB (*5*). During a systematic search for MDR TB among TB patients in Burkina Faso for whom a retreatment regimen was failing, we documented 2 cases of XDR TB.

## The Study

In 2006, a program was established by the National Tuberculosis Program, Burkina Faso; the University of Brescia, Italy; and the San Raffaele Scientific Institute, Italy, to perform drug-susceptibility testing (DST) on sputum samples from patients listed in the Burkina Faso Chronic TB Register. To be included in this register, patients must have experienced treatment failure after standard and retreatment regimens consisting of the following (in order): 2 months of streptomycin, rifampin, isoniazid, pyrazinamide, and ethambutol; then 1 month of rifampin, isoniazid, pyrazinamide, and ethambutol; and then 5 months of rifampin, isoniazid, and ethambutol. Failure was defined as a persistently positive sputum smear after 5 months of treatment. All patients were informed and consented to the study.

From January 2006 through March 2009, a total of 156 patients with chronic TB were registered in Burkina Faso. For 88 patients, sputum samples were collected before treatment with second-line drugs; for 48, they were collected at 1–12 months of treatment with second-line drugs. Samples were immediately frozen and stored at –20°C before being transferred on dry ice for culture and first-line DST at the University of Brescia and for second-line DST and genotyping at San Raffaele Scientific Institute. Samples were cultured on an MGIT 960 automated system (Becton Dickinson Microbiology Systems, Cockeysville, MD, USA) according to the manufacturer’s instructions. DST to first- and second-line drugs was performed on all *Mycobacterium tuberculosis* isolates by classic dilution method.

*M. tuberculosis* was isolated from 50 samples; 45 patients had not yet taken second-line drugs and 5 had. Of the 50 isolates, 34 (68%) were confirmed as MDR TB, 29 from untreated patients and 5 from treated patients. We identified 2 cases of XDR TB (5.9% of all MDR TB cases): 1 patient was sampled at month 24 of treatment with second-line drugs, and 1 was initially classified as having MDR TB before his disease progressed to XDR TB during follow-up treatment with second-line drugs.

Patient 1 was a 33-year-old man who was born in Côte d’Ivoire, moved to Burkina Faso in 2000, and received a diagnosis of smear-positive pulmonary TB in July 2003. He received an 8-month standard treatment regimen (including 6 months of continuation with isoniazid and ethambutol). Treatment was directly observed during the first 2 months. In January 2004, his treatment was classified as failed, and he immediately started a standard retreatment regimen. Because his sputum did not clear by month 5, the patient was registered as a patient with chronic TB. During 2004, he traveled throughout Mali and Côte d’Ivoire. Back in Burkina Faso in January 2005, he was admitted to the reference national hospital for patients with chronic TB. His HIV test result was negative. At this time neither culture and DST nor a standard second-line drug regimen approved by the Green Light Committee were available in Burkina Faso. The patient was empirically prescribed kanamycin, ethionamide, ciprofloxacin, and pyrazinamide, which he took under direct observation as a hospital inpatient for 21 months; at discharge, his sputum samples remained positive.

In December 2006, 2 sputum samples showed resistance to all first-line drugs; resistance to second-line drugs amikacin, ofloxacin, ethionamide, and cycloserine; and susceptibility to para-aminosalicylic acid and capreomycin. Appropriate and effective drugs were unavailable in the country. The patient was readmitted to the ward for patients with chronic TB and placed in a single isolation room. He died of TB in August 2008. Known contacts were monitored closely. A housemate was listed in the Chronic TB Register in June 2006. He traveled from Burkina Faso to Israel before DST could be performed. The patient’s sister-in-law died of MDR TB in March 2007; DST confirmed resistance to all first-line drugs but susceptibility to amikacin and ofloxacin.

Patient 2 was a 44-year-old man. His MDR TB was diagnosed in Côte d’Ivoire in June 2007, and he immediately moved back to Burkina Faso to seek care. His HIV test result was negative. Sputum samples collected in July 2007 grew MDR *M. tuberculosis* resistant to amikacin, kanamicin, ethionamide, and closerine but sensitive to ethambutol and ofloxacin. In September 2007, the patient started treatment with Green Light Committee–approved second-line drugs (6 months of pyrazinamide, ofloxacine, kanamycin, ethionamide, and cycloserine followed by 15 months of ofloxacin, ethionamide, and cycloserine). Treatment was directly observed during the first 13 months. His sputum samples remained positive throughout treatment, and additional DST in October 2008 showed an XDR strain of *M. tuberculosis*. Mycobacterial interspersed repetitive unit genotyping showed an identical pattern for strains detected initially and during follow-up.

## Conclusions

Our findings confirm that XDR TB can be found wherever the search capacity exists. Thus, despite unavailability of evidence for the widespread existence of drug resistance, high priority should be given to strengthening laboratory capacities in sub-Saharan Africa (*6*). Also, because XDR TB developed while patient 2 was receiving second-line TB drugs, optimal adherence during intensive and continuation phases of second-line treatment regimens should be ensured. Staff should receive specific training with regard to managing the frequent side effects.

Our study had 1 major limitation. Because our sample was a select population and included patients already receiving treatment with second-line drugs, we may have overestimated the proportion of XDR TB cases among MDR TB cases.

Mechanisms of emergence of XDR TB in Burkina Faso differ from those in South Africa, where most identified XDR TB cases are primarily resistant, occur among HIV-infected patients, and result from exposure in the hospital or the community (*7*). Because each of the 2 XDR TB patients in Burkina Faso reported long-term stays in neighboring countries, we believe that response to the MDR TB challenge should be based on regional rather than national strategies.

Our study supports current policies for strictly controlled introduction of second-line drugs and the current Green Light Committee strategy that requires demonstration of compliance with guidelines for proper management of MDR TB before granting access to second-line drugs (*8*). Stakeholders and TB program managers in Africa should be reminded that the main reasons for development of resistance in TB are poor patient adherence to treatment regimen, inappropriate drug prescription, irregular drug supply, and poor drug quality (*9*). When these factors occur with use of second-line TB drugs, the result will be XDR TB.

Rapid adoption of a programmatic approach to management of MDR TB (*6*) is warranted in Burkina Faso and probably other countries in western Africa. In countries without effective programs for community or outpatient care of patients with MDR TB, hospital care in reference centers enables appropriate follow-up during the initial phases of treatment. In this context, implementation of appropriate infection control measures should rank high among program priorities (*10*).

## References

[1] Kwon YS, Kim YH, Suh GY, Chung MP, Kim H, Kwon OJ, Treatment outcomes for HIV-uninfected patients with multidrug-resistant and extensively drug-resistant tuberculosis. Clin Infect Dis. 2008;47:496–502. 10.1086/590005 18611154

[2] Gandhi NR, Moll A, Sturm AW, Pawinski R, Govender T, Lalloo U, Extensively drug-resistant tuberculosis as a cause of death in patients co-infected with tuberculosis and HIV in a rural area of South Africa. Lancet. 2006;368:1575–80. 10.1016/S0140-6736(06)69573-1 17084757

[3] World Health Organization. Countries with XDR-TB confirmed cases as of June 2008 [cited 2009 Aug 18]. http://www.who.int/tb/challenges/xdr/xdr_map_june08.pdf

[4] World Health Organization. Global tuberculosis control: surveillance, planning, financing. WHO report 2009. WHO/HTM/TB/2008.411. Geneva: The Organization; 2008.

[5] World Health Organization. WHO anti-tuberculosis drug resistance in the world. Fourth global report. WHO/HTM/TB/2008.394. Geneva: The Organization; 2008.

[6] World Health Organization. Guidelines for the programmatic management of drug-resistant tuberculosis. Emergency update 2008. WHO/HTM/TB/2008.402. Geneva: The Organization; 2008.

[7] Andrews JR, Gandhi NR, Moodley P, Shah S, Bohlken L, Moll AP, Exogenous reinfection as a cause of multidrug-resistant and extensively drug-resistant tuberculosis in rural South Africa. J Infect Dis. 2008;198:1582–9. 10.1086/592991 18847372

[8] Gupta R, Cegielski JP, Espinal MA, Henkens M, Kim JY, Lambregts-Van Weenzembeek CS, Increasing transparency in partnerships for health—introducing the Green Light Committee. Trop Med Int Health. 2002;7:970–6. 10.1046/j.1365-3156.2002.00960.x 12390604

[9] Espinal MA, Laserson K, Camacho M, Fusheng Z, Kim SJ, Tlali RE, Determinants of drug-resistant tuberculosis: analysis of 11 countries. Int J Tuberc Lung Dis. 2001;5:887–93. 11605880

[10] Chen-Yuan C, Enarson DA, Fujiwara PI, Van Deun A, Jen-Jyh L. Strategies of extensively drug- resistant TB risk management for health workers and other care givers. Expert Review of Respiratory Medicine. 2008;2:47–54 [cited 2010 Feb 25]. http://www.expert-reviews.com/toc/ers/2/1 10.1586/17476348.2.1.47 20477221

